# Genomic profile of Pancoast syndrome due to hepatocellular carcinoma: A case report

**DOI:** 10.1111/1759-7714.14923

**Published:** 2023-05-09

**Authors:** Drew T. Bergman, Lorraine Zaki, Jason R. Pettus, Bassem I. Zaki, Manik Amin

**Affiliations:** ^1^ Geisel School of Medicine at Dartmouth Hanover New Hampshire USA; ^2^ Dartmouth‐Hitchcock Medical Center Lebanon New Hampshire USA; ^3^ University of Rochester Rochester New York USA; ^4^ Pathology and Laboratory Medicine Dartmouth‐Hitchcock Medical Center Lebanon New Hampshire USA; ^5^ Radiation Oncology Dartmouth‐Hitchcock Medical Center Lebanon New Hampshire USA; ^6^ Hematology/Oncology Dartmouth‐Hitchcock Medical Center Lebanon New Hampshire USA

**Keywords:** hepatocellular carcinoma, immunotherapy, palliative radiation, Pancoast tumor, superior sulcus tumor

## Abstract

Hepatocellular carcinoma (HCC) is a common cancer and is frequently diagnosed at a late and unresectable stage with limited effective treatment options. Here, we present the fifth reported case of a 77 year‐old male with metastatic HCC presenting as a symptomatic superior sulcus lung tumor and discuss the genomic profile of this rare presentation of HCC for the first time, which included multiple classic mutations in HCC such as *TERT*, *TP53*, and *WNT*/β‐catenin signaling as well as in the DNA repair gene *ATM*. The patient was treated with palliative radiotherapy to the Pancoast tumor followed by atezolizumab plus bevacizumab and passed away 6 months after diagnosis. This rare case highlights the need for effective treatment in aggressive and unresectable HCC and the utility of early genomic studies to allow for targeted therapy such as poly (ADP‐ribose) polymerase (PARP)‐inhibitors.

## Graphical abstract

We report a rare case of metastatic hepatocellular carcinoma presenting as a symptomatic Pancoast tumor and discuss its genomic profile, which included classic mutations such as the *TERT* promoter, *TP53*, and the *WNT*‐signaling pathway, as well as in *ATM*, a DNA repair pathway member targetable by PARP inhibition. This case highlights a strange presentation of Pancoast syndrome, the need for effective treatment in advanced HCC, and the utility of early genomic studies to allow for targeted therapy.

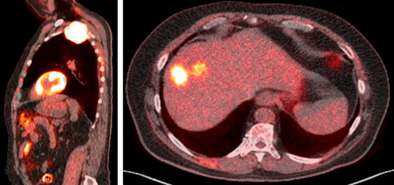

## INTRODUCTION

Superior pulmonary sulcus tumors are almost exclusively primary non‐small cell lung cancer (NSCLC) in origin and can present with shoulder pain, Horner syndrome, upper arm edema, and weakness and decreased sensation in the upper extremity.^1^ Hepatocellular carcinoma (HCC) is the most common cause of liver cancer, most commonly secondary to hepatitis B or C infection and cirrhosis.^2^ It is frequently diagnosed at an advanced unresectable stage with metastasis, commonly in the lung. Current standard of care for unresectable HCC is immune checkpoint inhibition with targeted therapy, yet nearly 60% of patients have disease progression or succumb to the disease.^3^ Here, we present a male patient with symptomatic superior sulcus tumor due to metastatic HCC, an exceedingly rare site of metastasis with few known cases reported in the literature.^4^, ^5^, ^6^, ^7^

## CASE REPORT

A 77 year‐old man with a history of myocardial infarction, diabetes mellitus, and cigarette smoking presented to the emergency department with 2 months of left shoulder pain, axillary swelling, left hand neuropathy, weight loss, fatigue, dyspnea on exertion, and dizziness. On examination he was afebrile, hypertensive, had decreased breath sounds in his left upper lobe, and palpable left axillary lymphadenopathy. There was decreased left grip strength and decreased sensation along the C7, C8, and T1 distributions. There was no miosis, ptosis, or anhidrosis.

**FIGURE 1:**
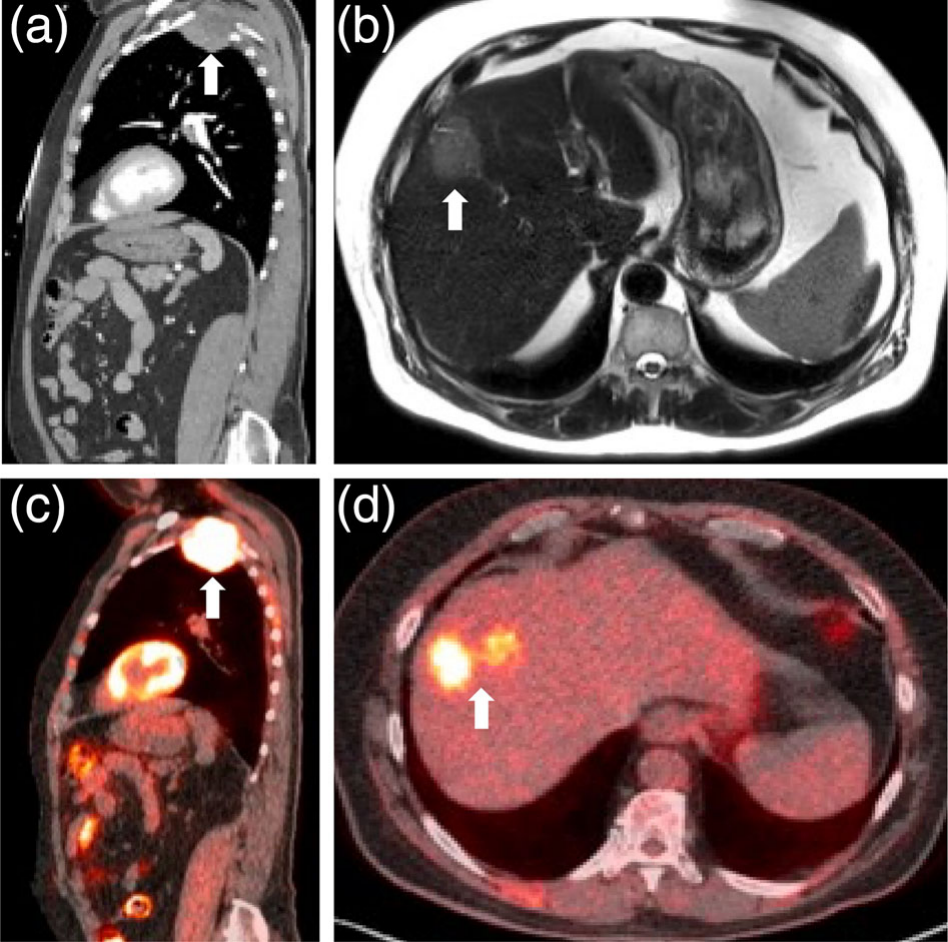
Imaging. (a) Initial sagittal computed tomography (CT)‐angiogram demonstrating a superior sulcus tumor (arrow). (b) T2W axial image demonstrating a mildly hyperintense mass (arrow) in the right hepatic lobe. (c) Sagittal staging positron emission tomography (PET)‐CT image confirming an F‐fluorodeoxyglucose (FDG)‐avid left apical mass (arrow) with interval growth. (d) Axial staging PET‐CT image confirming a lobulated heterogeneously FDG‐avid mass (arrow) in the anterolateral right hepatic lobe.

**FIGURE 2:**
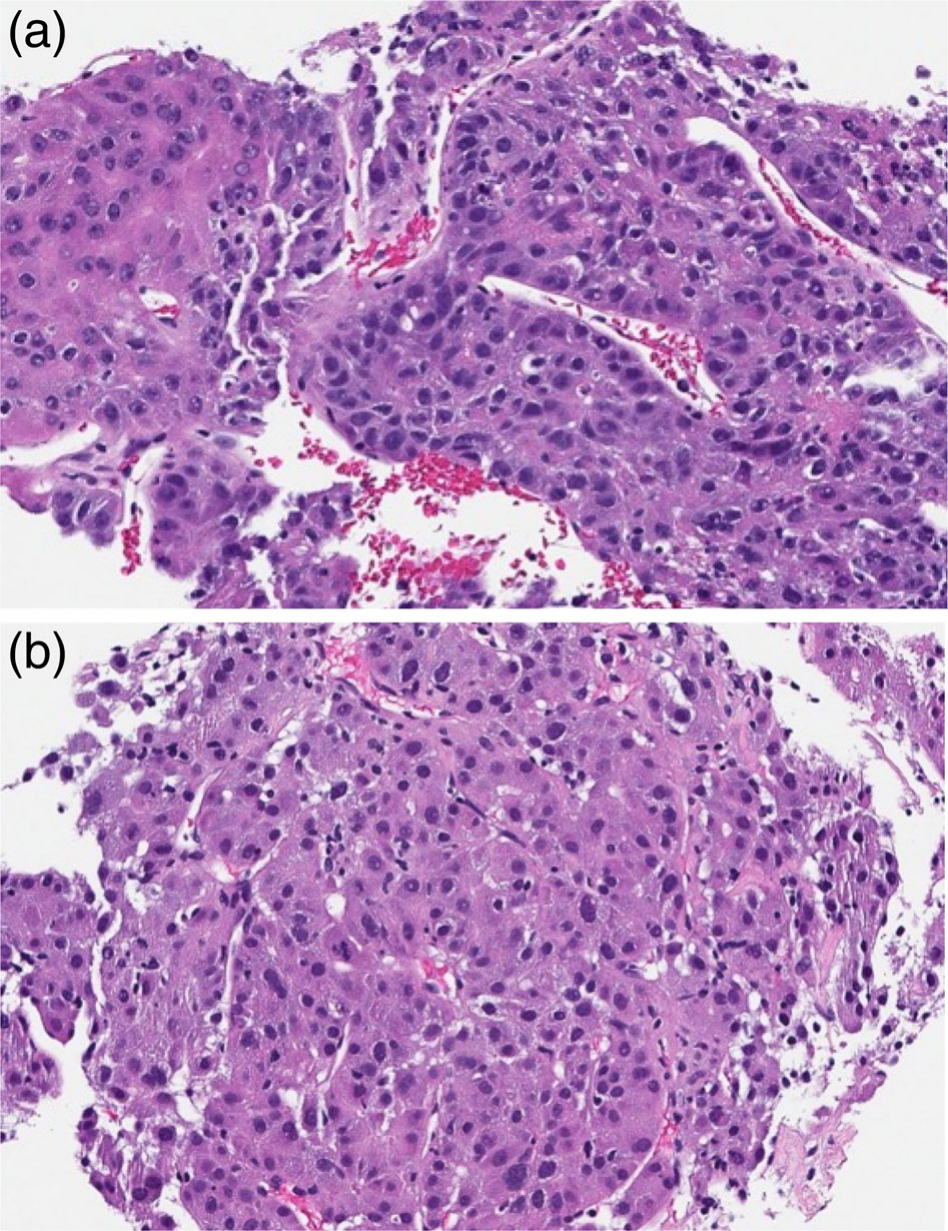
Pathology of primary liver mass and Pancoast tumor. (a) Lung biopsy showing moderate‐poorly differentiated metastatic hepatocellular carcinoma involving pulmonary parenchyma (hematoxylin and eosin [HE], digitally scanned at 40x resolution). (b) Liver biopsy showing moderate‐poorly‐differentiated primary hepatocellular carcinoma, similar in morphology to the metastatic lung tumor (HE, digitally scanned at 40x resolution).

Chest computed tomography (CT) showed a 5.6 cm left apical lung mass with chest wall invasion and lytic osseous erosion and a solitary 4.5 cm liver mass (Figure 1). Magnetic resonance imaging (MRI) confirmed these findings (Figure 1). Subsequent liver mass biopsy showed moderate to poorly differentiated HCC (Figure 2). This result prompted biopsy of the Pancoast tumor, which was immunophenotypically and morphologically consistent with metastatic HCC (Figure 2). Both neoplasms displayed immunohistochemistry positivity for arginase‐1 and hepatocyte paraffin antigen‐1 (HepPar‐1). The lung tumor was also negative for p 40 and TTF1.

Anti‐HCV and HBsAg were negative, α‐1 antitrypsin was at the upper limit of normal, and the α‐1 antitrypsin S and Z alleles were not detected. The serum α‐fetoprotein (AFP) level was highly elevated at 22891 ng/mL. Staging PET‐CT scan showed a 6 cm F‐fluorodeoxyglucose (FDG)‐avid lobulated liver mass and a 7 cm FDG‐avid Pancoast tumor invading the left chest wall, T2/T3 vertebral bodies, and spinal canal. There were multiple new subcentimeter bilateral pulmonary nodules and lytic lesions in the anterolateral left fourth rib and T7 vertebral body consistent with metastasis (Figure 1).

Both the primary liver tumor and lung metastasis underwent targeted tumor somatic mutation testing in a CLIA‐certified laboratory (Foundation Medicine, Inc, CDx panel) that assesses for substitutions, insertion and deletion alterations, and copy number alterations in 324 cancer‐related genes, as well as select gene rearrangements. This panel showed the following variants in both tumors: ataxia telangiectasia mutated (*ATM*) rearrangement in exon 51, *CTNNB1* (K355T), *TERT* promoter (‐124C > T), and *TP53* (G334V) mutations. In addition to these findings, the left apical lung metastasis also showed an *STK11* loss. Specimen tumor content was inadequate to assess for microsatellite status (MS) and tumor mutation burden (TMB) in the liver primary tumor; however, the lung tumor was MS‐stable with a TMB of 4 Muts/Mb. No other genes commonly implicated in HCC had detectable alterations (Table 1).

**TABLE 1:** Mutations by disease site.

Gene	Site	
	Liver	Lung
*TERT* promoter	−124C > T	−124C > T
*TP53*	G334V	G334V
*CTNNB1*	K335T	K335T
*ATM*	exon 51^a^	exon 51^a^
*STK11*	Wild‐type	Deletion

^**a**^ Rearrangement in *ATM* exon 51.

The patient received palliative 3D conformal radiotherapy (RT, 37.5 Gy in 15 fractions) to the Pancoast tumor, during and after which his pain was relatively stable. Palliative systemic therapy was then initiated with bevacizumab and atezolizumab^3^ which unfortunately was complicated by proteinuria (≥ 300 mg/dL)^8^ so he continued on atezolizumab alone for the subsequent cycle. Two weeks later he was hospitalized with increasing dyspnea, fatigue, nausea, dehydration, and weight loss. A chest CT showed interval progression of his disease, including increased size of both the lung and liver lesions. After discussion of goals of care and further treatment options, he received sorafenib chemotherapy as a second‐line therapeutic but was only able to complete 4 days of treatment after developing fatigue, nausea, and diarrhea. After further discussion he decided to discontinue all systemic therapy and focus on quality of life. He was enrolled in home hospice and died 6 months after diagnosis.

## DISCUSSION

HCC is a common, deadly cancer usually associated with hepatitis B or C infection and liver cirrhosis. It often presents late in the disease course, limiting the ability for resection. The current first‐line therapy for unresectable and advanced HCC, atezolizumab plus bevacizumab, still leads to progression or death in 60% of cases, highlighting the need for further breakthroughs in treatment.^3^ The etiology of Pancoast tumors is almost exclusively bronchogenic carcinoma. Although HCC frequently metastasizes to the lung, it has only been known to manifest as a Pancoast tumor in four other reported cases.^4^, ^5^, ^6^, ^7^ These cases similarly report the initial presentation of Pancoast syndrome, a tissue diagnosis of primary HCC with metastatic HCC in the superior sulcus, highly elevated alpha fetoprotein (AFP), and poor response to treatment.

This is the first reported case of metastatic HCC Pancoast tumor in 15 years. Recent advances in the genomics of HCC have improved our understanding of the oncogenic events in this disease, allowing us to report the genomics of this unique form of HCC for the first time. This patient's primary HCC and Pancoast tumor had identical mutations in three of the most common genes and pathways mutated in HCC, including the *TERT* promoter, *TP53*, and *CTNNB1* (*WNT*‐signaling), providing additional confirmation that the Pancoast tumor was indeed metastatic HCC and explaining in part the patient's aggressive disease course and poor response to treatment.^9^ This genomic profile is further unique in that simultaneous *TP53* and *CTNNB1* mutation is rare in HCC (~6% of patients).^10^ These canonical pathways mutated in HCC are all potentially targetable. Telomerase is an attractive target in many cancers with potential methods including immunotherapies, direct telomerase inhibitors, as well as targeting *TERT* gene expression driven by *TERT* promoter mutation.^11^ Promising methods to target *TP53* under clinical investigation include reactivation of mutant p53 protein with APR‐246 and COTI‐2 and inhibiting the interaction between p53 and MDM2/MDM4.^12^ *WNT*‐signaling, and targeting *CTNNB1* specifically, is currently under clinical investigation: napabucasin (BBI608, NCT02279719), PRI‐724 (NCT02195440), and sulindac.^13^

Deletion of the tumor suppressor serine threonine kinase 11 (*STK11*) was detected only in the superior sulcus metastasis and not in the primary tumor. *STK11* is involved in the activation of PI3K/Ras signaling, another commonly activated pathway in HCC.^14^ This led us to speculate whether the development of this additional proliferative signal allowed the patient's tumor to invade the superior sulcus of the left lung.

The largest potential for the addition of current targeted therapy in this patient's case was treatment of the rearrangement of *ATM* with poly (ADP‐ribose) polymerase (PARP) inhibition. ATM is a PI3K‐related serine/threonine protein kinase involved in genomic integrity and DNA repair that is commonly associated with germline mutations and increased risk for malignancy and has been reported to be somatically mutated in HCC.^15^ DNA repair pathways are upregulated in HCC, in part because of altered expression of *PARP1*.^16^ PARP inhibitors, such as the FDA‐approved olaparib, are FDA‐approved to treat *BRCA*‐mutated breast, ovarian, and pancreatic cancer and have shown promise in clinical trials for solid tumors with *ATM* loss of function and in models of HCC, although more work is yet to be done to identify patients with mutations that impact ATM activity as the mutational profile is heterogenous.^17^, ^18^ While clinical data is currently limited, durable response was achieved using olaparib and cisplatin in another case of advanced HCC with mutation in a similar DNA damage response gene *FANCA*.^19^

Emerging systemic treatments for advanced HCC that have shown promise in clinical trials include new multikinase inhibitors (donafenib), new immune checkpoint inhibitor monotherapy (durvalumab), and new combinations of checkpoint inhibitors (durvalumab plus tremelimumab).^20^ This case underscores the importance of further development of regimens to improve the yet dismal prognosis of advanced unresectable HCC and the utility of early genomics studies to enable targeted therapy.

## AUTHOR CONTRIBUTIONS

All authors contributed to conceptualizing the case, writing, and editing the mansucript.

## FUNDING INFORMATION

This research did not receive any specific grant from funding agencies in the public, commercial, or not‐for‐profit sectors.

## CONFLICT OF INTEREST STATEMENT

The authors have no conflicts of interest to disclose.

## INFORMED CONSENT

Informed consent for this case report was acquired.
